# Hypothyroidism Induced Hyponatremia: A Case Report

**DOI:** 10.31729/jnma.7471

**Published:** 2022-09-30

**Authors:** Gyabina Maharjan, Mandeep Kumar Yadav, Nirajan Khati, Shailendra Kumar Yadav, Tulsi Ram Shrestha

**Affiliations:** 1 Kirtipur Hospital, Devdhoka, Kritipur, Nepal; 2 Nepal Bharat Maitri Hospital, Mitrapark, Kathmandu, Nepal; 3 Vayodha Hospital, Balkhu, Kathmandu, Nepal; 4 Ishan Children and Women's Hospital, Basundhara, Kathmandu, Nepal

**Keywords:** *case reports*, *hyponatremia*, *hypothyroidism*, *myxedema coma*

## Abstract

Thyroid hormones have various effects on the body which include electrolyte and water hemostasis. It is also involved in renal development and physiology. Hyponatremia is a serious electrolyte imbalance that can be associated with the involvement of different body systems and a wide range of deleterious changes. We report a case of a 62-years-old female with the symptoms of severe hyponatremia like altered mental sensorium with a serum sodium level of 110 meq/l. After ruling out other causes, a final diagnosis of hypothyroidism was made. On treating hypothyroidism the symptoms of hyponatremia were resolved. Therefore, thyroid stimulating hormone determination is mandatory during the evaluation of hyponatremia. And, those patients should be treated with fluid restriction and treatment of hypothyroidism.

## INTRODUCTION

Thyroid hormones have various effects on the body, which include electrolyte and water hemostasis. It is also involved in renal development and physiology.^1^ Hypothyroidism has been historically implicated in the development of hyponatremia. Severe untreated hypothyroidism or acute stressors like infection, and myocardial infarction can lead to a life-threatening condition known as myxedema coma.^2^ A diagnosis of myxedema coma is rare and mostly presents in elderly females with typical progression in lethargy leading to stupor.^3^ Hence, we describe a case of a 62-years-old female with the symptoms of severe hyponatremia like altered mental sensorium with a serum sodium level of 110 meq/l.

## CASE REPORT

A 62 years female presented with symptoms of altered mental sensorium for 1 day to the local Emergency department (ED). According to the patient's relative, she had been feeling tired with difficulty performing daily life activities, decreased appetite, easy fatigability, increased sensitivity to cold along with puffy face, and dry skin. Due to her poor socioeconomic condition, the patient had never been investigated for thyroid functions although she had symptoms of hypothyroidism for a long time. In addition, her chest infection precipitated her condition leading to a myxedema coma. On further evaluation and ruling out other causes of hyponatremia, the patient's condition was likely to be associated with an increase in thyroid stimulating hormone level. The initial physical examination triage revealed a Glasgow Coma Scale (GCS) of E2V2M4, with a respiratory rate of 18 bpm; 98% oxygen saturation in room air; blood pressure of 110/70 mmHg; a pulse of 69 bpm; and a temperature of 98.6°F on axillary armpit. Also, the random blood sugar level was 167 mg/dl. The patient was hyponatremic (Serum sodium level: 110 meq/l), his urine osmolality was 236.5 mosm/kg and his plasma osmolality was 248 mosm/kg. The free T3 level, T4 level and TSH levels were 0.91 pg/ml, 0.11 ng/dl and 171.50 mIU/l respectively. After an initial assessment of the patient, arterial blood gas (ABG) analysis was done that showed respiratory acidosis with severe hyponatremia and hypocalcemia. After stabilisation of the patient in the ED, the patient was shifted to intensive care unit (ICU) for further management.

When hypothyroidism was treated the level of sodium showed improvement. After ruling out various causes in this patient such as adrenal insufficiency, syndrome of inappropriate antidiuretic hormone secretion (SIADH), and primary polydipsia, we came to know that hypothyroidism is the main cause of hyponatremia in this patient.

## DISCUSSION

Quality of life has been affected as the general population has a high prevalence of thyroid disease which can cause serious abnormalities and disorders.^4^ Hyponatremia is a serious, electrolyte imbalance that can be associated with the involvement of different body systems and a wide range of deleterious changes Thus, there are substantial challenges in managing this disorder due to its various etiologies and comorbidities associated with hyponatremia.^5,6^ A rare but possible cause of hyponatremia would be a low thyroid hormone level.^7^ The pathogenesis behind the low sodium level in hypothyroidism is explained in two ways: firstly, there is a decrease in the delivery of water in the distal nephron due to excess secretion of vasopressin which impairs water excretion in the setting of hypothyroidism.^1^ Secondly, hypothyroidism also leads to a reduction in glomerular filtration rate secondary to thyroid hormone deficiency on cardiac output and peripheral vascular resistance.^8-10^

However, a treating physician should also include other differential diagnoses in mind which include adrenal insufficiency, syndrome of inappropriate antidiuretic hormone, and primary polydipsia.^11^ One should first need to rule out these differential diagnoses before concluding that hypothyroidism is the cause of hyponatremia.^6,7^ Therefore, TSH hormone determination is mandatory during the evaluation of hyponatremia. And, those patients should be treated with fluid restriction and treatment of hypothyroidism.^8^

We have concluded that the majority of the cases of Hyponatremia are related to primary hypothyroidism. Extreme TSH level like in our case has been directly linked to being the cause of low sodium level. Therefore, after ruling out other causes of hyponatremia we can conclude that hypothyroidism is one of the causes of hyponatremia.
